# Editorial: Natural substances: A new weapon against antibiotic-resistant bacteria in the clinical and veterinary field

**DOI:** 10.3389/fmicb.2022.987615

**Published:** 2022-07-29

**Authors:** Ramona Iseppi, Carla Sabia

**Affiliations:** Department of Life Sciences, University of Modena and Reggio Emilia, Modena, Italy

**Keywords:** antibiotic-resistant bacteria, essential oils, antimicrobial peptides, bacteriocins, clinical infections

Antimicrobial resistance is a critical public health problem. The increasing emergence of antibiotic-resistant bacteria and multidrug-resistant bacteria can be attributed to the misuse of antibiotics and the absence of effective antibiotics on the market. Antimicrobial resistance is a trouble in both human and veterinary medicines. This problem has led to a “One Health” approach to coordinate efforts and slow the development of drug-resistant microorganisms. In veterinary medicine, antibiotics are used to treat clinical infections and, in some countries, also as antimicrobial growth promoters. Antibiotic resistance in the veterinary field has led to a limited use of antimicrobials specifically for human health, making it essential to develop alternatives for feed production. Therefore, there is a need to search for new antibacterial compounds and new strategies to fight bacterial infections. In research for alternative therapies, essential oils (EOs), bacteriocins, or antimicrobial peptides (AMPs) have been shown to be effective against a wide range of bacterial species, including multidrug-resistant bacteria. The rise of antimicrobial resistance and the antibiotic crisis has expanded research into the potential use of EOs, bacteriocins, and AMPs in the biomedical field for the prevention or treatment of infectious diseases. In addition, these compounds can be applied in all fields related to veterinary practice, have proven useful in the control of foodborne pathogens in foods of animal origin (preventively), as well as in potential clinical therapeutic use to fight infections in animal species. Combining these alternative substances of natural origin (EOs, bacteriocins, and AMPs) with antibiotics has been recognized as a strategy to improve the therapeutic effect and limit the development of antibiotic resistance.

As part of this multi-strategy approach, the purpose of this Research Topic was to provide a platform for the publication of up-to-date information and high-quality research on antibiotic resistance control strategies. Two articles focused on the control of biofilms produced by methicillin-resistant *Staphylococcus aureus* and *Staphylococcus epidermidis*. Yu et al. isolated and identified four flavonoid-type analog compounds from *Woodfordia uniflora* that inhibited biofilm formation in methicillin-resistant *S. aureus* (MRSA) and synergized with methicillin. The metabolite analysis revealed that this synergizing activity by compounds was achieved by remodeling metabolism including central carbon metabolism and glutamine biosynthesis that causes an imbalance in the flow of precursors for biosynthesis of both bacterial cell envelopes and biofilm formation of MRSA.

Similarly, Qian et al. studied the potential antibacterial activity of 2-methoxycinnamaldehyde (MCA), a compound in cinnamon, against methicillin-resistant *S. epidermidis* (MRSE). The authors found that MCA was able to inhibit both the proliferation and biofilm formation of MRSE, indicating that MCA might not only affect the growth of MRSE but also inhibit the pathogenic potential of this microorganism. SEM and TEM showed that MCA caused morphological changes and leakage of DNA, RNA, and cellular contents of MRSE. Furthermore, the authors noticed that the metabolic flux through the pentose phosphate pathway (PPP) was over-regulated accompanied by elevated ROS production which may be partially due to the increased metabolic flux through the TCA cycle.

New bacteriocins and substance such as bacteriocins have been partially characterized. In particular, the bacteriocin biosynthetic potential of the psychrophilic *Clostridium estertheticum* complex (CEC) through genome mining was determined by Wambui et al. The authors analyzed 40 CEC. The genome analysis determined the presence of 20 bacteriocin biosynthetic gene clusters (BBGC) encoded in plasmids in 18 out of the 40 genomes. A specific screening linked two BBGC encoding a lantibiotic and sactipeptide, respectively, with activity against *Bacillus cereus*, while other two lantibiotic encoding were linked with activity against *B. cereus, S. aureus* (methicillin-resistant), *Escherichia coli*, and *Pseudomonas aeruginosa*. The mass spectrometry (MS/MS) analysis revealed that CEC produces cesin A, a short natural variant of nisin, and a novel sactipeptide named estercticin A.

Fernández-Fernández et al. tested the production of bacteriocin-like inhibitory substances (BLIS) on a collection of 890 staphylococci of different origins (human, animal, food, and environment) and species, both coagulase-positive (CoPS) and coagulase-negative (CoNS). A genetic characterization (by PCR and sequencing) led to the identification of the 60 BLIS+ isolates produced in the highest percentage by CoNS staphylococci. This highlights the ability of ConNS, generally regarded as common colonizers and non-pathogenic bacteria, to be able to compete with other bacteria, including pathogens.

Another alternative approach toward antibiotic-resistant bacteria might be the biological production of nanoparticles. In light of this, a marine actinobacterium strain *Streptomyces catenulae* M2, isolated from marine water, was used by Khalil et al. to produce biosynthesized silver nanoparticles (Bio-SNPs). Bio-SNPs alone and in combination with Piperacillin-tazobactam showed an antibacterial activity against multidrug-resistant (MDR) bacteria. Nanoparticles combined with β-lactamase inhibitors revealed a synergism effect resulted from a binding interaction between antibiotic molecules that contained hydroxyl and amino groups that could easily react with Bio-SNPs.

Instead, Scandorieiro et al. evaluated the antibacterial activity of binary combinations containing bioAgNP (biogenically synthesized silver nanoparticles using *Fusarium oxysporum*), oregano essential oil, carvacrol, and thymol. The combination containing bioAgNP and oregano derivatives, in particular thymol, shows a strategic antibacterial mechanism; thymol disturbs the selective permeability of the cell membrane and consequently facilitates the access of the nanoparticles to the bacterial cytoplasm. The results of this study highlight the potent action of *F. oxysporum*-bioAgNP combined with carvacrol or thymol against Gram-negative bacteria, including carbapenem-resistant strains such as *E. coli, Klebsiella pneumoniae, Acinetobacter baumannii*, and *P. aeruginosa* which have become a major concern in hospitals. Another approach to combat infections caused by antibiotic-resistant microorganisms is the use of native antimicrobial peptides (AMPs), which are one of the essential effector molecules in many life forms, including fish, to combat microbial tissue invasion.

Okella et al. identified, fractionated, and characterized seven potential AMPs from African catfish's skin mucus. The antimicrobial activity of AMPs against *S. aureus* ATCC 25923 and *E. coli* ATCC 25922 can, among other reasons, be attributed to their low molecular weight (between 1,005 and 1,622 kDa).

Therefore, this Research Topic shows that the search for new antibacterial compounds and new strategies to fight multidrug-resistant bacterial infections is a challenging field of research. We are confident that the manuscripts included in this Research Topic will inform the scientific community about the current knowledge and challenges that have yet to be overcome on alternative therapies for the prevention or treatment of infectious diseases.

## Author contributions

All authors listed have made a substantial, direct, and intellectual contribution to the work and approved it for publication.

## Conflict of interest

The authors declare that the research was conducted in the absence of any commercial or financial relationships that could be construed as a potential conflict of interest.

## Publisher's note

All claims expressed in this article are solely those of the authors and do not necessarily represent those of their affiliated organizations, or those of the publisher, the editors and the reviewers. Any product that may be evaluated in this article, or claim that may be made by its manufacturer, is not guaranteed or endorsed by the publisher.

